# HIV-1 and Human *PEG10* Frameshift Elements Are Functionally Distinct and Distinguished by Novel Small Molecule Modulators

**DOI:** 10.1371/journal.pone.0139036

**Published:** 2015-10-08

**Authors:** Tony S. Cardno, Yosuke Shimaki, Brad E. Sleebs, Kurt Lackovic, John P. Parisot, Rebecca M. Moss, Caillan Crowe-McAuliffe, Suneeth F. Mathew, Christina D. Edgar, Torsten Kleffmann, Warren P. Tate

**Affiliations:** 1 Department of Biochemistry, University of Otago, Dunedin, New Zealand; 2 The Walter and Eliza Hall Institute of Medical Research, Parkville, Australia; 3 Department of Medical Biology, University of Melbourne, Parkville, Australia; 4 Centre for Protein Research, University of Otago, Dunedin, New Zealand; University of British Columbia, CANADA

## Abstract

Frameshifting during translation of viral or in rare cases cellular mRNA results in the synthesis of proteins from two overlapping reading frames within the same mRNA. In HIV-1 the protease, reverse transcriptase, and integrase enzymes are in a second reading frame relative to the structural group-specific antigen (gag), and their synthesis is dependent upon frameshifting. This ensures that a strictly regulated ratio of structural proteins and enzymes, which is critical for HIV-1 replication and viral infectivity, is maintained during protein synthesis. The frameshift element in HIV-1 RNA is an attractive target for the development of a new class of anti HIV-1 drugs. However, a number of examples are now emerging of human genes using −1 frameshifting, such as *PEG10* and *CCR5*. In this study we have compared the HIV-1 and *PEG10* frameshift elements and shown they have distinct functional characteristics. Frameshifting occurs at several points within each element. Moreover, frameshift modulators that were isolated by high-throughput screening of a library of 114,000 lead-like compounds behaved differently with the *PEG10* frameshift element. The most effective compounds affecting the HIV-1 element enhanced frameshifting by 2.5-fold at 10 μM in two different frameshift reporter assay systems. HIV-1 protease:gag protein ratio was affected by a similar amount in a specific assay of virally-infected cultured cell, but the modulation of frameshifting of the first-iteration compounds was not sufficient to show significant effects on viral infectivity. Importantly, two compounds did not affect frameshifting with the human *PEG10* element, while one modestly inhibited rather than enhanced frameshifting at the human element. These studies indicate that frameshift elements have unique characteristics that may allow targeting of HIV-1 and of other viruses specifically for development of antiviral therapeutic molecules without effect on human genes like *PEG10* that use the same generic mechanism.

## Introduction

Currently, 33 million people are estimated to be infected with HIV-1 globally, with an annual death rate estimated to be 1.8 million [1]. Although now regarded as a chronic disease in developed countries, in part due to the availability of combination therapies that contain a cocktail of different drug classses, HIV-1 infection still remains a significant ongoing health problem with an increasing occurrence of drug resistance in patients to one or more of the drugs in current use [2]. In developing countries it remains a persistent threat to populations not able to access expensive drugs and, in particular, the virus continues to cause devastation in sub-Saharan Africa, Asia and Eastern Europe.

Currently, there are four different life cycle stages of HIV that are targeted therapeutically: (i) fusion and entry of the virus to the host cell (fusion inhibitors), (ii) reverse transcription of viral RNA into DNA (reverse transcriptase inhibitors), (iii) integration of the viral DNA into the host genome (integrase inhibitors) and (iv) protease digestion of HIV polyproteins (protease inhibitors). Patients harboring drug resistant strains of virus are a growing subgroup that highlights the need for new treatment options. One strategy to keep the therapeutic options ahead of the ability of the virus to develop resistance to available drugs is to develop new combination therapies focusing on a broader target spectrum [3].

HIV, along with a variety of other retroviruses such as simian immunodeficiency virus (SIV), feline immunodeficiency virus (FIV), and coronaviruses like SARS, use a rare genetic recoding mechanism called programmed translational frameshifting or programmed ribosomal frameshifting to regulate the expression of key genes [4–6]. In HIV, two major genes, *gag* and *pol* overlap by 205 nucleotides [7]. The downstream *pol* gene that encodes the protease, reverse transcriptase and integrase within a single polypeptide does not have its own initiation codon and is in a different reading frame (−1) relative to gag. A change in the reading frame (or frameshift) is required for the encoded information for these proteins to be translated. This leads to a gag-pol fusion polyprotein being synthesized dependent upon a −1 frameshifting event. Efficient programmed translational frameshifting requires two elements within the mRNA: a highly conserved slippery sequence (U UUU UUA) [8], and a downstream RNA stem-loop that enhances the efficiency of −1 frameshifting [7].

The synthesis of the HIV-1 gag-pol fusion protein is tightly regulated, and occurs only once in every ten to twenty passages of the host ribosome (i.e. at a 5–10% efficiency) across a frameshift element in the viral mRNA [9]. Genetic variations in the frameshift element that reduce frameshifting by as little as 3-fold (that is, decrease the rate from 5–10% down to 2–3%) have profound defects on viral replication [10], and even more modest changes can reduce viral replication [11]. Conversely, viral infectivity is also dramatically decreased if the frameshift element is modified to significantly enhance frameshifting instead of the 5–10% seen with the native element [12–13].

When interest in −1 frameshifting mechanism as a potential drug target first arose there were no documented examples of specific human genes (or indeed any genes in the animal kingdom) that used the mechanism in their expression, but the human genome contains an evolutionary history of retroelement insertions, and there was potential for frameshifting in those now domesticated as functional human genes. Indeed the human developmental gene *PEG10* (*paternally expressed gene 10*) undergoes −1 frameshifting [14]. Important for placental development [15], *PEG10* is expressed 10 days post- conception in mouse placenta and amniotic membranes. Synthesis of the protein has not been found in adult tissues [14] but preliminary evidence has been obtained that the protein is also expressed in the hypothalamus [16], and the adrenal glands [17]. However, as yet we have no definitive evidence for frameshifting [16]. Another poorly characterized family of 15 human genes, *MA/Pnma*, has been determined to have retroelement ancestry, and two members have the potential for frameshifting, *Ma3* and *Ma5*. The *Ma3* frameshift element has been shown to support 20% frameshifting *in vitro* [18] but to date no evidence has been presented for its occurrence *in vivo*, or what function it might be supporting. Also more recently, the chemokine receptor *CCR5* has been identified as a human gene that utilizes −1 frameshifting for fine tuning the regulation of its expression [19]. The new reading frame leads to a premature stop codon, initiating nonsense-mediated decay for destabilization of the mRNA. *CCR5* frameshifting is stimulated by endogenous microRNAs, emphasizing the importance of screening in cells *in vivo* where endogenous modulators are functioning.

*PEG10*, *Ma3*, and *CCR5* have evolved with different slippery sequences and quite different secondary structural elements to HIV-1, and therefore it may be possible to identify compounds that are specific to only the HIV-1 frameshift element. To identify compounds that modulate HIV frameshifting, several large screens have previously been undertaken. For example, a screen of 56,000 compounds by Ribogene Inc. identified a compound RG501 (also known as DB213) that enhanced frameshifting leading to a 50% decrease in viral replication *in vitro*, although this compound was not a suitable candidate for use as a therapeutic agent [20]. More recently *in vitro* assays have shown that RG501 bound to and stabilized the RNA stem-loop, characteristic of the HIV-1 M group frameshift elements [21].

A diverse library of approximately 11,000 compounds, termed “resin-bound dynamic combinatorial chemistry (RBDCC)”, was screened for their binding affinity to labeled HIV-1 regulatory stem-loop RNA [22]. This work was expanded to further analogues to show Structure Activity Relationship (SAR) for their RNA binding affinities [23]. The goal was to find molecules that specifically bound with high affinity and stabilized or de-stabilized the HIV-1 stem-loop RNA, leading to changes in frameshift efficiency. More recently, compounds that enhanced frameshifting by more than 50% were shown to decrease infectivity in a single-round model infectivity assay by >90% at 20 μM [24].

Potential frameshift-modulating compounds against a human virus must be evaluated against the frameshift sites of human genes that use this mechanism. A picture is emerging whereby each frameshift site might have specific features for their regulation. In this study we investigate this by comparing specific aspects of the mechanism of frameshifting in HIV-1 and *PEG10*, utilizing a class of modulator compounds that affect the HIV-1 frameshift element that we have identified through a high-throughput screen, and on which we are now undertaking further study.

## Materials and Methods

### Chemical Library

The WEHI chemical library consisted of 114,000 lead-like compounds selected from a variety of vendors to give a diverse set of molecules. Compounds with more than 85% similarity were excluded from the library based on a Tanimoto coefficient along with other filters as previously described [25]. The compounds were stored at Compounds Australia (Griffith University, Brisbane, QLD) in DMSO under a controlled atmosphere.

### Construction of reporter vectors

The dual luciferase or bifluorophore reporter vectors were constructed with a Gateway® (Invitrogen) cloning cassette placed between two reporters. This enabled the rapid cloning of genes of interest, with the reporter gene upstream of the frameshift element acting as an internal control to normalize output data between different assay wells and plates [26]. The sequence of interest inserted between the reporters, whether it was an HIV-1 frameshift element or variant of it, or a nonsense codon readthrough element, regulated the expression level of the second reporter and was used to assess either frameshifting or stop codon readthrough.

Two Gateway® (Invitrogen) compatible plasmid destination vectors were constructed, an EGFP-RfA-tdTomato plasmid and a hRLuc-RfA-Luc^+^ plasmid. For the GFP related destination vectors a Gateway® (Invitrogen) Reading Frame (Rf) Cassette A (RfA) was ligated into an EcoRV site between the two fluorphore reporters as described in [27]. However making the dual luciferase vector required additional steps. The reporter gene hRLuc has an internal 0-frame EcoRV site (GATATC) that was replaced by the synonymous mutation GACATT using PCR mutagenesis. PCR was then used to retain the 5′ HindIII and replace the 3′ stop codon with a new EcoRV site. The start codon of the firefly Luc^+^ reporter gene (Promega) was replaced with a BamHI site, and further modified by introducing a NotI site 3′ of the stop codon, to enable cloning into the plasmid backbone.

Four different gene sequences were cloned into the destination vectors using BP clonase (Invitrogen): two HIV frameshift elements, (i) the wild-type slippery sequence U UUU UUA followed by GGG and the extended stem-loop [28], and (ii) a variant U UU*C CU*A that does not support frameshifting. Additionally, an adenine nucleotide (A) cloned immediately 5′ of the GGG placed the downstream reporter in the 0-frame so each reporter would be in the same frame and be produced in an equimolar ratio (used to normalize the outputs of the assay and calculate frameshift efficiency). Two sequences from the cystic fibrosis chloride channel *CFTR* were cloned, the W1282X human nonsense mutant gene sequence containing a UGA stop codon [29], and a 0-frame laboratory constructed control where the UGA was replaced with a UGG sense codon.

The dual luciferase reporter vectors were stably integrated into Flp-In™-293 cell lines (Invitrogen). For some experiments the dual fluorophore reporter vectors were transiently transfected into cells as the fluorescence output from stably integrated GFP reporters was sufficient to give a robust assay in a 96 well format but not in 384-well formats.

### Stable cell lines expressing luciferase reporters

Dulbecco’s modified Eagle’s medium (DMEM) and hygromycin B were purchased from Invitrogen (Carlsbad, CA). Fetal bovine serum (FBS) was purchased from Thermo Fisher Scientific (Melbourne, VIC, Australia).

The stable Flp-In™-293 reporter cells (Invitrogen) were maintained in DMEM supplemented with 10% heat-inactivated FBS and incubated in a humidified environment at 37°C in 5% CO_2_. Cells in phenol red- free DMEM were seeded at 5000 cells/well using 384-well white μCLEAR plates (Greiner Bio-One) and incubated with compound at 37°C with 5% CO_2_. To obtain an accurate background signal, control wells were seeded with cells that were incubated with 1 mM cycloheximide to inhibit all protein synthesis.

### Transient expressing cells with GFP reporters

Cells (HEK 293T) were transfected and immediately seeded into 24-well plates. A transfection reagent (a mixture of serum-free DMEM and Xtreme GENE HP DNA transfection reagent (Roche)) was incubated at room temperature, before it was added drop-wise in a ratio of 2:1 to dual fluorophore (EGFP/tdTomato) plasmid DNA for the transfection. The vectors contained either the HIV-1 frameshift element or the modified 0-frame control. Cells were then added to the Xtreme GENE/DNA mix, and plated. Lead compounds were then added to the wells and cells allowed to grow and divide for 48 h at 37°C, 5% CO_2_. The media was then aspirated from the wells, the cells washed with PBS, and lysed with Passive Lysis buffer (Promega). Plates were rocked for 15 min at room temperature (Stuart Scientific Platform STR8) and the lysate was stored overnight at −20°C.

### Luciferase Reporter gene detection

The luciferase reporter genes were detected using the Dual-Glo® Luciferase Assay System (Promega) using an Envision 2103 multi-label plate reader (Perkin Elmer) with a 384-well luminescence aperture.

### Fluorophore Reporter gene detection

For fluorescence detection, lysates were thawed and transferred to a 96-well black plate (Greiner Bio-One). Plates were analysed in a POLARStar optima (BMG LABTECH). EGFP fluorescence was detected with 485 nm excitation/520 nm emission. The tdTomato fluorophore was detected with 544 nm excitation/590 nm emission.

### Firefly luciferase inhibition assay

Compounds were incubated with 5 nM of QuantiLum® Recombinant Luciferase (Promega) for 10 min, before luciferase activity was measured using the Dual-Glo® Luciferase Assay System (Promega) with an Envision 2103 multi-label plate reader (Perkin Elmer) with a 384-well luminescence aperture.

### Data Analysis

#### Z′-factor

The Z′-factor was calculated for the cell-based assay as it provides a tool for comparing and evaluating quality of an assay [30]. A Z′-factor of ≥0.5 represents a robust assay amenable for high-throughput screening.

#### Percentage activity

The percentage activity comprised of a firefly (Luc^+^) and a *Renilla* (hRLuc) read of the same well was calculated as:

$$Activity\;\%=100\times \frac{L_{firefly}-{\mu LN}_{firefly}}{L_{Renilla}-{\mu LN}_{Renilla}}$$

#### Percentage efficiency

The percentage efficiency normalized for any off-target compound activity against the assay reporters. This was achieved by running duplicate compound plates that were seeded with the 0-frame cell line.

Efficiency%=100×Lfirefly−μLNfireflyLRenilla−μLNRenillaLfirefly0frame−μLNfirefly0frameLRenilla0frame−μLNRenilla0frame

#### Control firefly luciferase activity

The control firefly luciferase activity was used as a measure of compounds effect on the firefly luciferase reporter.

$$Control\;firefly\;activity\;\%=100\times \frac{L_{firefly_{0frame}}-{\mu LN}_{firefly_{0frame}}}{L_{Renilla_{0frame}}-{\mu LN}_{Renilla_{0frame}}}$$

Where: *L*_firefly_ = Luminescence of firefly reporter

*L*_*Renilla*_ = Luminescence of *Renilla* reporter

μ_LNfirefly_ = Mean luminescence of firefly reporter treated with cycloheximide

μ_LNfirefly_ = Mean luminescence of *Renilla* reporter treated with cycloheximide

*L*_firefly 0frame_ = Luminescence of firefly reporter in the 0-frame

*L*_Renilla 0frame_ = Luminescence of *Renilla* reporter in the 0-frame

μ_LNfirefly 0frame_ = Mean luminescence of firefly reporter in 0-frame treated with cycloheximide

μ_LNfirefly 0frame_ = Mean luminescence of *Renilla* reporter in 0-frame treated with cycloheximide

### Strategy for detecting positions of frameshifting within HIV-1 and *PEG10* frameshift elements

Primers were designed for insertion of the HIV-1 and *PEG10* frameshift elements into a pMALc2 vector adding a C-terminal hexahistidine (His) tag in the −1-frame. A stop codon was introduced into the 0-frame just after the stem-loop in the HIV-1 element. This meant 138 nucleotides were inserted with a stop codon in the –1-frame immediately following His tag sequence. Translation products contained the amino acids of the *MalE* gene and the amino acids of the HIV-1 frameshift element in the 0-frame until a stop codon was reached (49.6kD), or where frameshifting occurred switching to the –1 frame and terminating with the His tag (46.8kD). In the case of the *PEG10* element, sequences included the slippery sequence and the pseudoknot as predicted by Manktelow et al 2005 [31], together with sequence encoding the His tag in the –1-frame followed by a stop codon. Translation products contained the amino acids of the *MalE* gene and the amino acids encoded by the *PEG10* frameshift element; 43.9 kD where the translation continues in the 0-frame until a stop codon is reached, or 46.4 kD where frameshifting has occurred terminating after the His tag.

Cultures (500 mL) of *Escherichia coli* transformed with the recombinant pMal vectors, were induced for expression of the MalE fusion proteins with 1 mM IPTG at A_600_ of 0.5 and grown overnight at 18°C. Pelleted cells were ground with alumina for 15 min, mixed with buffer (20 mM Tris-HCl pH 8.0, 200 mM NaCl, 1 mM EDTA, 1 mM DTT, 1 mM PMSF). The supernatant obtained after centrifuging at 10,000 x g was mixed with prewashed amylose beads for 1 h at 4°C with rotation to bind the MalE proteins. After washing with the above buffer the MalE proteins were eluted with the same buffer containing 10 mM maltose. After dialysis into 50 mM Na_2_HPO_4_/NaH_2_PO_4_ pH 8.0, 300 mM NaCl, 5 mM imidazole the solution was mixed with prewashed Talon (Co^2+^) resin for 1 h at 4°C with rotation to bind the –1 frameshift products containing the His tag. After washing in the same buffer His-tagged proteins were eluted with in the same buffer, containing 250 mM imidazole, and then they were dialysed in buffer without imidazole. Samples were separated on a 14.5% polyacrylamide gel prior to analysis by mass spectrometry.

### Mass spectrometry identification of sites of frameshifting

Peptides containing the site of frameshifting were identified by nanoflow uHPLC-coupled tandem mass spectrometry as described previously [32]. In brief protein bands containing either the PEG10 or HIV-1 frameshift products were excised and in-gel digested with trypsin [33]. Tryptic peptides were then separated by nanoflow RP-uHPLC on an in-house packed emitter tip column (75 μm ID silica tubing packed with 3 μm C18 beads at a length of 12 cm) in line coupled to the nanospray source of a LTQ Orbitrap XL mass spectrometer (Thermo Scientific, San Jose, CA) using a gradient developed from 5% solvent B (0.2% formic acid in acetonitrile) in solvent A (0.2% formic acid in water) to 30% solvent B in solvent A over 12 min, followed by an increase to 95% solvent B in solvent A over 6 min. The LTQ Orbitrap mass spectrometer was operated in data dependent mode to allow for the acquisition of one full MS spectrum in the Orbitrap analyzer at a resolution of 60,000 (at m/z 400) and 5 ion trap CID fragment ion spectra per cycle. Raw data was processed through the Proteome Discoverer software using default settings. Spectra were then searched against a combined amino acid sequence database containing all SwissProt/UniProt sequence entries (546,057 entries) and 8 custom-made sequences covering four predicted sites of frameshifting for HIV-1 and *PEG10* using the Mascot (http://www.matrixscience.com/server.html) and SequestHT (Thermo Scientific, San Jose, CA) search engines. The searches were set up for tryptic peptides including the variable modifications of oxidized methionine, carboxyamidomethyl cysteine and deamidated asparagine and glutamine. For both search engines the Percolator algorithm [34] was used to adjust the score threshold for significant peptide identification at a false discovery rate of <1%.

### Small modulators of frameshift efficiency of the HIV-1 element

To identify small-molecule modulators of HIV-1 −1 frameshifting we designed a screening platform that used a transgenic human cell line to translate the viral frameshift element. A cell line that expressed the frameshift element inserted between *Renilla* luciferase (hRLuc) and firefly luciferase (Luc^+^) reporter genes out of frame with each other was used in a high-throughput screen of 114,000 lead-like compounds. The hits identified from the screen were then confirmed utilizing a series of counter screens. A control construct, with the same reporter genes in-frame, and a modified element that eliminated frameshifting at the site, was used as a counter screen to eliminate those compounds that acted off-target, for example, by modulating the signal of the downstream Luc^+^ reporter. A second counter screen used a construct with a sequence element containing a known nonsense mutation of the cystic fibrosis chloride channel gene *CFTR*. This element was cloned between the same reporter genes to indicate whether inhibitory or stimulatory compounds were specific for the frameshift element or affected readthrough of nonsense mutations as well. We also tested effects on a purified Luc^+^ enzyme for a counter screen. An orthologous cell-based assay with fluorescent reporters was used to ensure effects of promising compounds were not related to the specific luciferase reporter assay. Finally we tested the effects of the most promising compounds on the frameshift efficiency of the human *PEG10* frameshift element, to determine their specificity for HIV-1.

### Effect of modulators on HIV-1 protein synthesis

To determine whether the enhancer compounds affected synthesis of HIV-1 proteins (in the 0-frame and –1-frame) with the same pattern and to the same degree as observed with the reporter constructs in the screening assay, synthesis of −1-frame protease relative to the 0-frame gag protein (P24) was determined in an HIV-1 infectivity assay. Firstly the compounds were tested for toxicity for the host H9 cells at increasing concentrations and at the three time points (24, 48 and 72 h) that were used for the HIV-1 infection experiments, comparing with untreated cells, or those treated with AZT as a control. For two compounds, A1 and A3, the results were within the normal variability expected and there was no significant cytotoxicity, whereas the other compound A2 showed >25% toxicity at the highest concentration and so was put aside for this study. Then H9 cells were either infected with HIV-1 IIIB or uninfected as a control, and treated with enhancer compounds A1 and A3 (at 1, 5 and 20 μM) and a DMSO vehicle solvent control (0 μM) and cells were harvested at 24 h, 48 h and 72 h for analysis by Western immunoblot of gag protein (P24) expressed in the 0-frame, with specific antibody followed by the appropriate secondary antibody/detection reagent. The filter was then stripped and re-probed with anti-GAPDH for lane-to-lane normalization of the protein extract and consistency of immunoblot development. On a separate gel the protease enzyme in the −1-frame was detected using an anti-protease antibody together with appropriate secondary antibody. The filter was stripped and re-probed as before with anti-GAPDH for lane-to-lane normalization of protein extracts and consistency of immunoblot development. The intensity of the developed bands was digitized and quantitated, and the frameshift efficiency calculated at each time point for each compound and at each concentration, and for the DMSO vehicle control. These analytical data were normalized to variations of the GAPDH intensities, and compared with the DMSO control.

## Results

**Fig 1:**
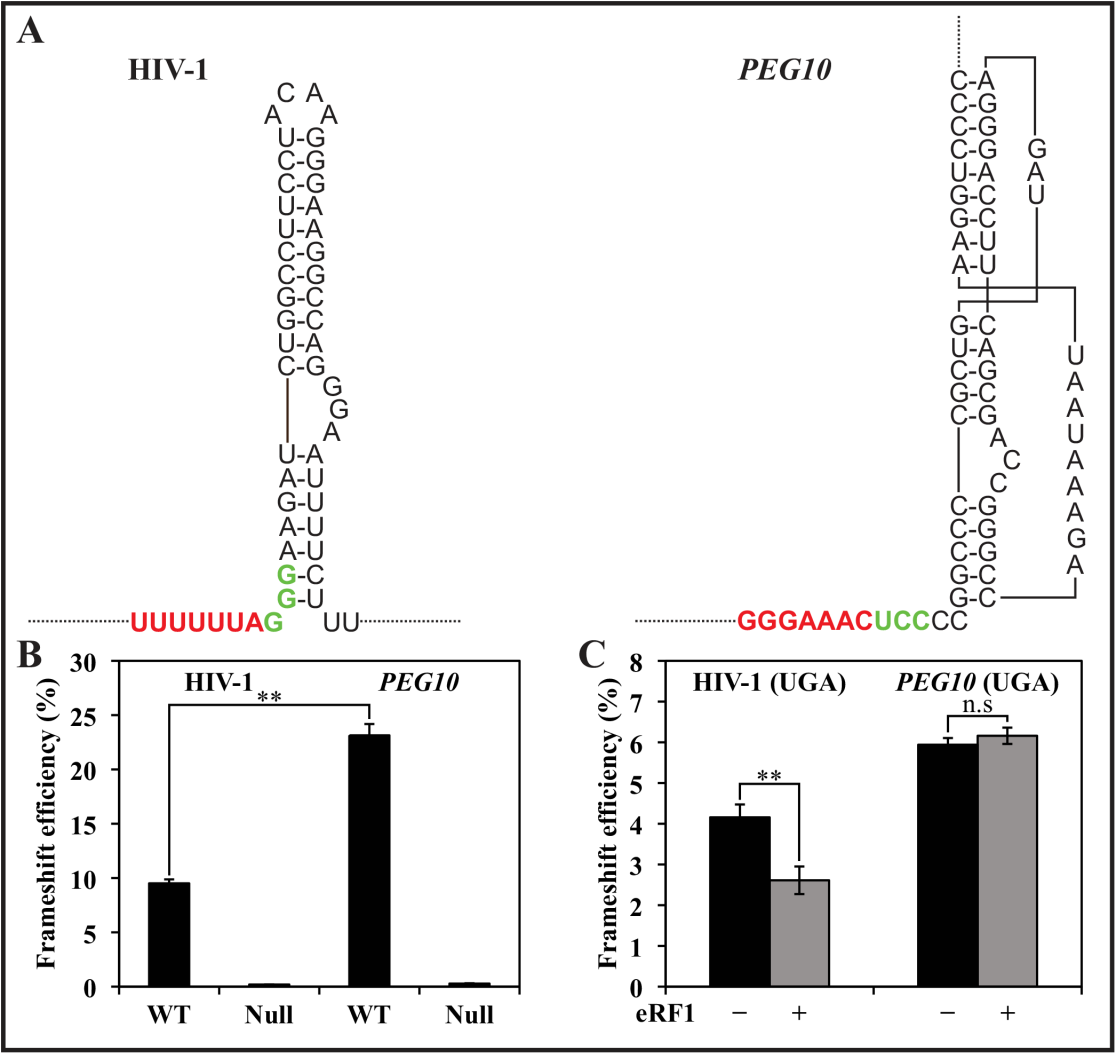
Comparison of frameshift activity within HIV-1 and human gene *PEG10* frameshift elements. (A) Structures of the HIV-1 and *PEG10* frameshift sites showing slippery sequence (red), intercodon (green) and secondary structural element (extended stem-loop and pseudoknot). (B) Frameshift efficiency at the elements within dual luciferase reporters (*Renilla* luciferase (hRLuc) upstream and firefly luciferase (Luc^+^ downstream) in the −1 frame. ‘WT’ refers to the native frameshift sequences shown above. ‘Null’ has the slippery sequence modified with mutations to nullify it as a frameshift element. (C) Frameshift efficiency of element with intercodon substitution ‘UGA’ has the GGG/UCC intercodons substituted with the stop codon UGA. Black bars are constructs with empty vector, and grey bars with ~3 fold overexpression of eRF1. Constructs were as described in [26] for HIV-1, and in [16] for *PEG10*. Assays were carried out as described in [16].

In the current study we have used luminescent and fluorescent dual reporters, both for the comparisons of the functional characteristics of the HIV-1 and *PEG10* frameshift sites, and for the screening of compounds affecting the HIV-1 frameshift element. For the comparison of the mechanisms operating at each site, we used transient transfection assays [26]. As shown in Fig 1A the HIV-1 and *PEG10* frameshift elements have unique features within a common structural background. Both have (i) slippery sequences (SS) of the form X XXY YYZ (U UUU UUA for HIV-1 and G GGA AAC for *PEG10*), (ii) different intercodons [26] (GGG and UCC respectively), (iii) distinct secondary structures (SStr) (extended stem-loop for HIV-1 and pseudoknot for *PEG10*), and (iv) the SStr start is within the intercodon in HIV-1, whereas it is two nucleotides downstream of the intercodon in *PEG10*. Perhaps with these differences it is not surprising that the frameshift efficiencies of the two elements are so different, as shown in Fig 1B (10% for HIV-1 and 22% for *PEG10*). In both cases, when the slippery sequences are modified to eliminate their homopolymeric nature, frameshifting is very low. If the highly conserved intercodon [26] is substituted with a stop codon frameshifting is dramatically reduced with both elements. For HIV-1, the efficiency can be further reduced by overexpressing the stop codon decoding molecule, eRF1 [26]. By contrast, overexpressing eRF1 had no further effect on frameshift efficiency with the *PEG10* element (Fig 1C). The constructs and assays utilized in these studies are as previously described [16,26].

**Fig 2:**
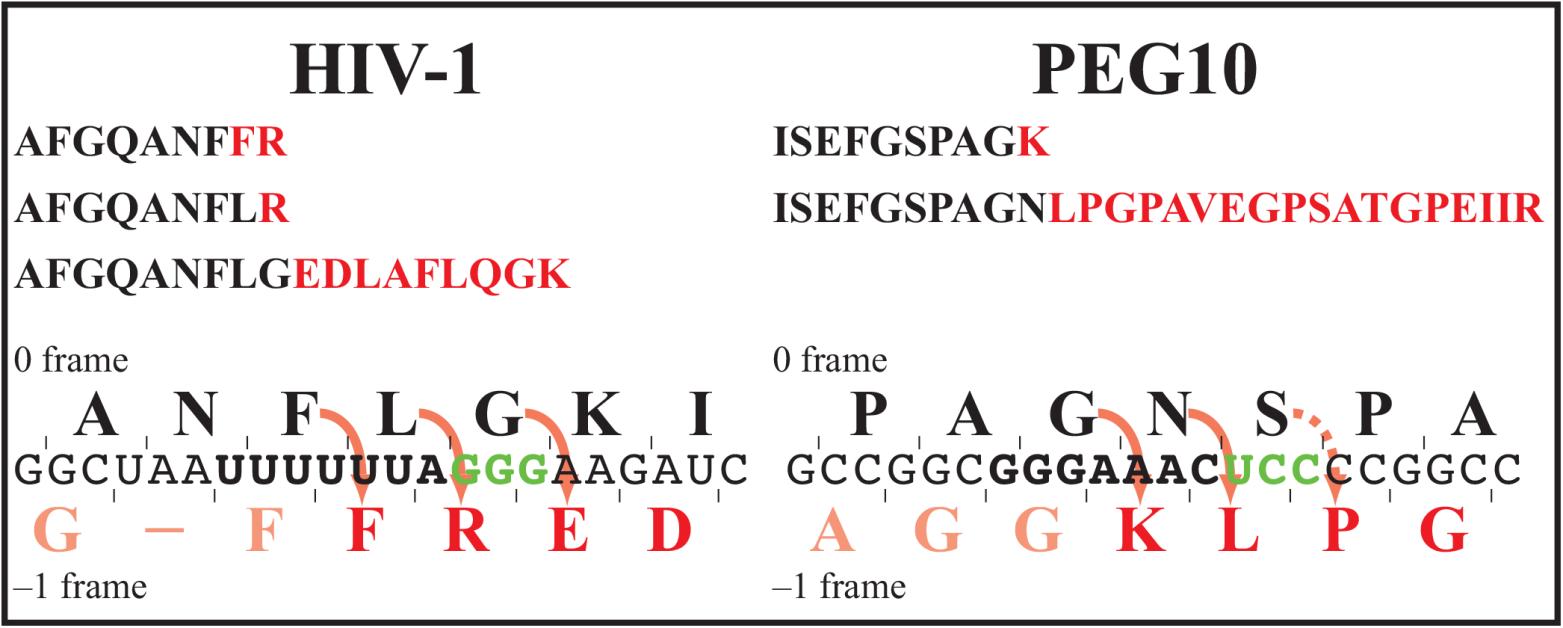
Identification of frameshift positions within the HIV-1 and *PEG10* frameshift elements. The peptides identified by tandem mass spectrometry are shown following translation of the HIV-1 and *PEG10* elements (see Table 1). Solid lines show the predicted positions of frameshifting within the sequences. The dashed line in the *PEG10* element indicates the detection of the predicted minor site of frameshifting following high resolution/accurate mass measurement of the respective tryptic peptide with a mass error of less than 1 ppm (0.0016 Da).

We then devised a platform that would allow the position of frameshifting within the elements to be identified so that the HIV-1 and *PEG10* sites could be compared. The frameshift elements were cloned behind a bacterial *MalE* gene and were followed by a His tag in the −1-frame. HIV-1 frameshifting has been shown to have the same characteristics on eukaryotic and bacterial ribosomes [26,35]. After expression of the constructs in *Escherichia coli* the expression products were isolated on amylose columns via binding of the maltose binding protein, and the frameshift products were then purified on Co^2+^ columns utilizing the −1-frame His tag. These products were subjected to mass spectrometry (MS)-based peptide identification (Table 1). In the case of HIV-1 four distinct peptides were identified, one a trypsin cleavage product of a larger peptide. Of particular interest, one such position contained the 0-frame glycine encoded by the intercodon, GGG. This peptide sequence implied decoding and frameshifting had occurred after GGG had been decoded, and explained why the overexpressed decoding molecule eRF1 as shown in Fig 1C had lowered further the frameshift efficiency when the GGG was substituted by a stop codon. For *PEG10* translation a contrasting result was obtained–three peptides were identified (one a fragment of a larger peptide Table 1). Their identities indicated frameshifting had occurred predominantly before the intercodon was encountered. There was however, evidence of a low abundance peptide that included the amino acid encoded in the UCC intercodon (Table 1). From the identification of these peptides the sites of frameshifting were deduced for each element and, in the case of *PEG10*, the minor site is indicated with a dotted arrow (Fig 2). The low level of the peptide arising from this event was consistent with a failure to detect significant modulation of *PEG10* frameshifting by decoding molecules with substitution of the intercodon by a stop codon (Fig 1C). As shown in Fig 1B, paradoxically this substitution alone still reduced frameshifting at the site specifically and substantially. This might result simply from peptidyl-tRNA fall-off as the ribosome is stalled or slowed at the site or as frameshifting is occurring since there is only one cognate re-pairing possible after slippage at the *PEG10* site in contrast to the two cognate re-pairings at the HIV-1 site. Such a fall-off would lower the production of the downstream reporter.

**Table 1:** Mass spectrometry identification of peptides containing the sites of frameshifting.

	Peptide	Charge	m/z	Error ppm	Sequest score	Mascot score	Peak area
PEG10							
	ISEFGSPAGK	2	496.7553	-1.52	2.24	*21	2.73E+06
	ISEFGSPAGNLPGPAVEGPSATGPEIIR	3	907.4691	1.34	6.71	106	1.38E+08
	ISEFGSPAGNSPGPAVEGPSATGPEIIR	3	898.7844	0.58	No CID	No CID	1.97E+05
	LPGPAVEGPSATGPEIIR	2	880.9817	1.06	4.82	83	6.18E+07
HIV							
	AFGQANFFR	2	529.2643	-0.03	2.69	59	1.39E+08
	AFGQANFLR	2	512.7653	2.08	2.84	57	4.27E+08
	AFGQANFLGEDLAFLQGK	2	963.493	1.62	6.01	89	5.84E+07
	EDLAFLQGK	2	510.7721	0.75	2.25	*39	1.30E+09

The charge state (Charge), measured m/z value (m/z) and error of measurement in parts per million (Error; ppm) are given for the strongest detected peptide signal for each of the predicted frameshifting events. The scores for peptide identifications are given for both the SequestHT and Mascot search engines. The Mascot spectrum to peptide sequence assignments marked by an asterisk were not considered high confidence identifications, but also did not match any other sequence in the SwissProt sequence database (546,057 sequence entries) and therefore represent the most likely spectrum to sequence match. The peptide intensity is given as the area under the curve (peak area) for the extracted ion chromatogram of the strongest peptide signal of each frameshifting event and can be used as a rough estimation of peptide abundance. No CID: this low intensity signal was not selected for collision induced dissociation tandem mass spectrometry. The peptide detection is therefore based on high mass accuracy precursor mass measurement with an error of <1 ppm (+/−0.0016 Da).

### A cell-based assay for screening of compounds that affect HIV-1 frameshifting

Results of the characterization of the HIV-1 and *PEG10* frameshift elements implied it might be possible to find compounds that would modulate frameshifting in HIV-1 but not with the *PEG10* gene. For the high-throughput screening, we developed a series of transgenic cell lines using the dual luciferase reporters (*Renilla*/firefly) and dual fluorescent reporters (GFP/tdTomato) created with Gateway® (Invitrogen) cloning cassettes. This enabled the cloning of the HIV-1 frameshift element, and expression of the downstream reporter indicated a frameshift rate within the expected range of 5−10% with slight variation depending on the reporters used [27,36]. Two HIV frameshift elements were constructed, the wild-type slippery sequence, U UUU UUA, followed by GGG and the extended stem-loop [28], and a variant U UU*C C*UA that does not support frameshifting. For an additional control (0-frame), an A was inserted immediately 3′ of the modified SS (ie U UU*C C*UA **A**), placing the downstream reporter in the 0-frame with respect to the upstream reporter. The cell lines expressing this sequence were used in counter screens to determine any off-target activity of the compounds being evaluated. The reporter constructs also contained a FlpIn™ (Invitrogen) integration site ensuring the reporter genes were stably integrated into the same genomic location so as to produce isogenic mammalian cell lines.

We elected to use a stepwise screening approach where we first screened 114,000 compounds at 10 μM from the curated WEHI library. The WEHI library consisted of diverse “lead-like” small molecules that possess synthetically amenable structures, are of high diversity as per Tanimoto dissimilarity analysis (T≤0.85), and are Lipinski compliant. The library had been filtered stringently to exclude any undesirable, reactive, promiscuous and assay-interfering compounds [25]. Luciferase reporters were used for the primary screen because of their high level of sensitivity that enabled frameshifting to be detected in stably transfected cells seeded in 384 well plates. The optimized 384 well assay using the Dual-Glo® Luciferase Assay System (Promega) enabled a high-throughput assay to be developed with a strong signal, with a background ratio of 10 and a Z´-factor of >0.5, indicating a robust platform for screening [30].

**Fig 3:**
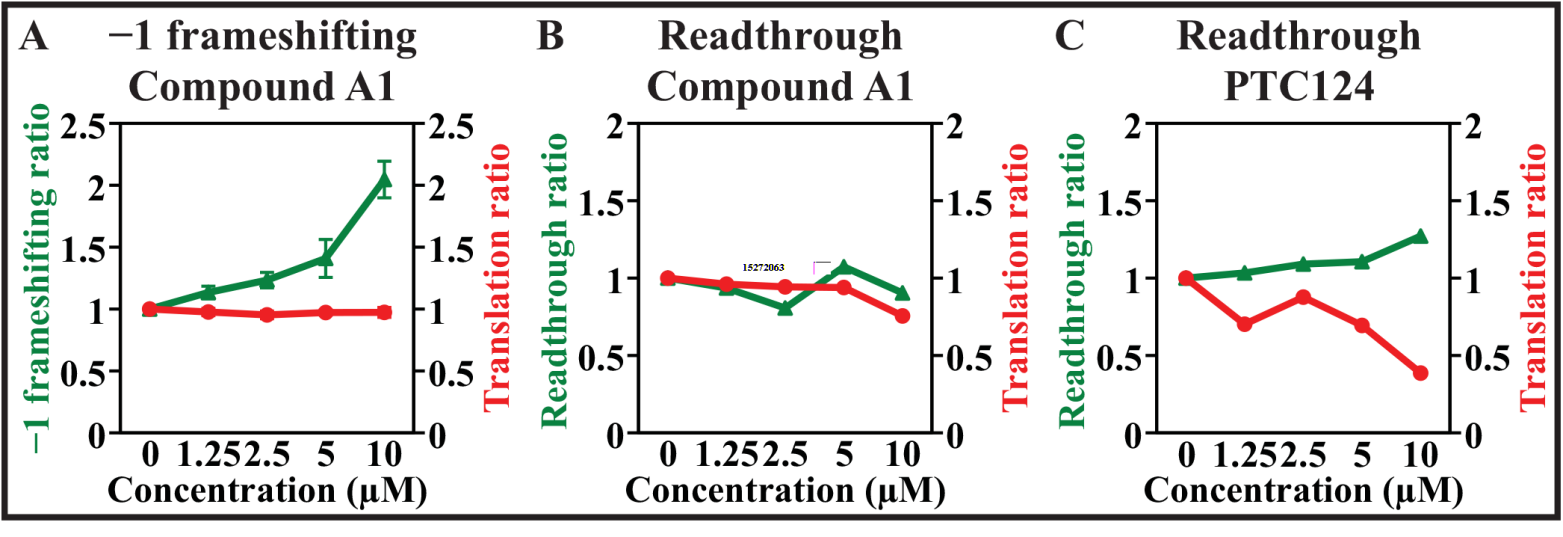
Frameshift enhancer A1 (A) evaluated in a dual luciferase based assay. Normalized frameshift efficiency of the HIV-1 element (green) is shown as −1 frameshift ratio relative to no compound (left ordinate). Effects of the compounds on the 0-frame control cell line containing the mutagenized slippery sequence is indicated in red, and is shown as translation ratio relative to no compound (right ordinate). (B) Compound A1 counter screen against a CFTR W1282X UGA nonsense mutant (green) and a UGG sense codon control (red). (C) PTC124 screened against CFTR W1282X (green) and the UGG sense codon control (red). Mean and standard deviation for three independent assays, each with n = 5 at 24 h.

We show here functional data with a frameshift enhancer that came through collective scrutiny of screens and counter screens. Frameshift enhancers like compound A1 showed increases in frameshifting with no apparent change in canonical protein synthesis up to 10 μM, as indicated by the upstream reporter (hRLuc) expression when the cells were assayed at 24 h after compound addition (Fig 3A green). The downstream reporter was not affected by these compounds when it was in the same frame as the upstream reporter (translation ratio 1.0) (Fig 3A red). This was reinforced separately by showing enhancer A1 did not modulate recombinant Luc^+^ enzyme activity. To ensure specificity for frameshifting, assays were employed to measure effects of the selected compounds on a different type of a genetic recoding site, the common UGA nonsense mutation W1282X found in the cystic fibrosis gene, *CTFR* [29]. As shown in Fig 3B enhancer A1 did not affect this different recoding event, either the readthrough of the UGA stop codon (green), or a UGG sense codon control at that site (red). As a positive control PTC124, the putative stop-codon readthrough enhancer, that is now known also to bind to and inhibit Luc^+^ enzymatic activity, was tested. The expected inhibition of Luc^+^ with increasing concentration of PTC124 [37] is reflected in Fig 3C (red) where there is an apparent decrease in the translation ratio of the sense codon control. This was confirmed directly by showing PTC124 decreased recombinant Luc^+^ activity. However, PTC124 enhanced readthrough of the UGA stop codon as expected when normalized against this sense codon control as shown in Fig 3C (green) and this effect was also dependent upon concentration. Collectively these data implied the enhancer A1 was a specific modulator of –1 frameshifting and did not affect the reporter enzymes or simply modulate ribosomal activity more generally.

**Fig 4:**
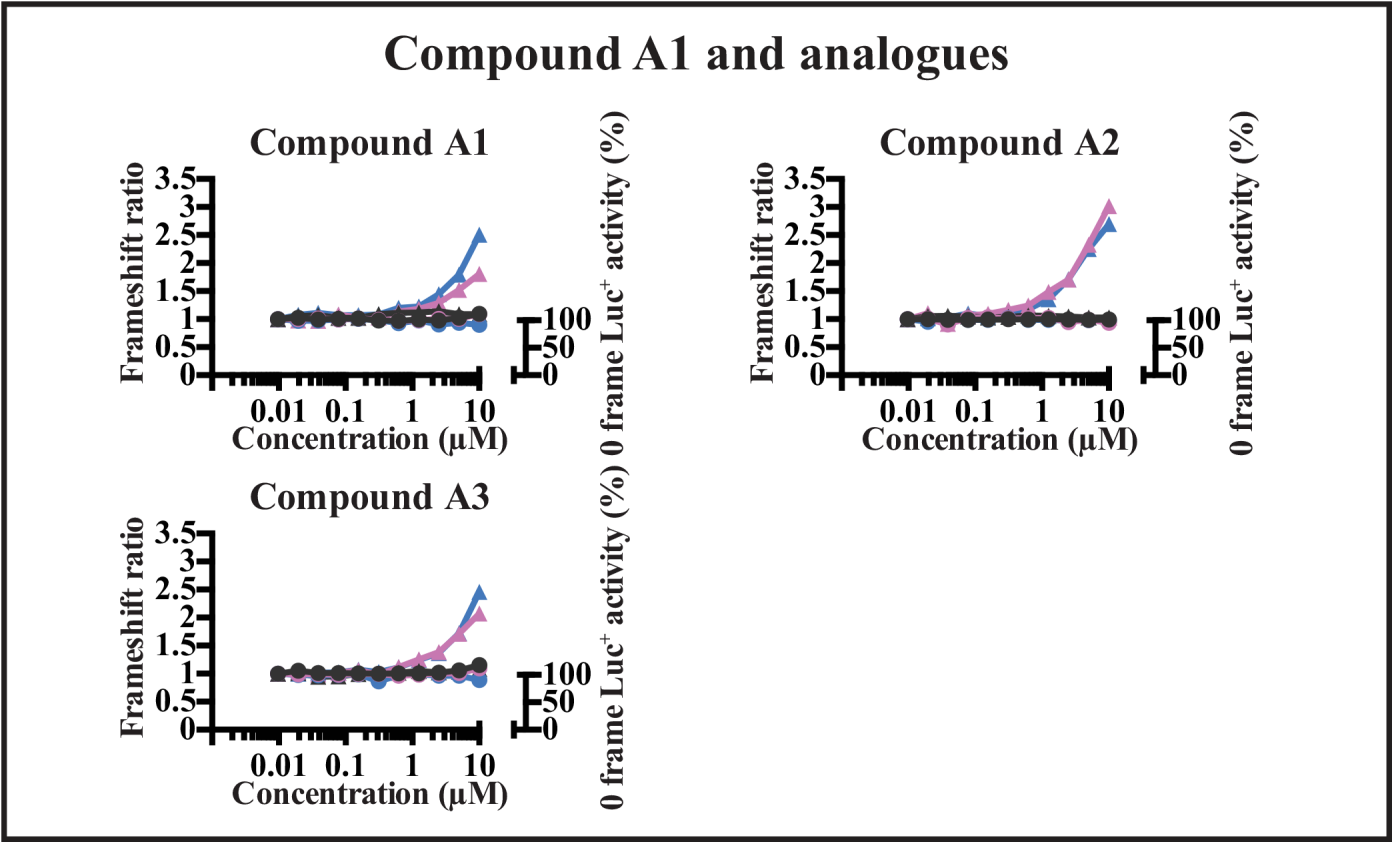
Effects on frameshifting at the HIV-1 element with increasing concentrations of enhancer A1 and two analogues A2 and A3 at three translational time points. The frameshift ratio of the cell line containing the HIV-1 frameshift element is indicated by triangles, as a comparison of that with no compound added. The firefly Luc^+^ activity in the control 0-frame cell-line is indicated by circles, and is expressed as the percentage of the activity with no compound. 0 h is indicated in black, 8 h in purple and 24 h in blue.

Frameshift enhancement by A1 and two analogues A2 and A3 were tested to ensure the enhancement was dependent upon translation. Analogue A2 was the most effective enhancer. Enhancement depended upon incubation (0–24 h, with 0, 8 and 24 h shown in different colors), and by increasing concentrations of the compounds (Fig 4). This indicated their effect required active translation. The activity of hRluc was unaffected by the compounds, and the enhancement effect was on the downstream reporter Luc^+^ output. By contrast, Luc^+^ output was not affected by any of the compounds when this reporter was in the 0-frame rather than the –1-frame (right hand scale), implying the enhancement seen was dependent upon shifting more translating ribosomes into the –1 frame, and not on modulation of the Luc^+^ activity itself as had been concluded from the studies of Fig 3.

### Do the compounds affect HIV-1 −1-frame enzyme protein production?

**Fig 5:**
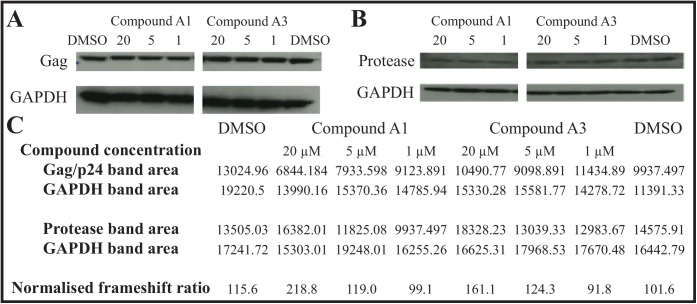
Enhancer compounds facilitate concentration-dependent increase in synthesis of −1-frame HIV-1 protease relative to the 0-frame gag protein. H9 cells, either infected with HIV-1 IIIB or uninfected as a control, were treated with enhancer compounds A1 and A3 (at 1, 5 and 20 μM) and a DMSO solvent control and cells were harvested at 48 h for analysis of protein expressed in the 0-frame, followed by the appropriate secondary antibody/detection reagent. The filter was then stripped and re-probed with anti-GAPDH for lane-to-lane normalization (A). On a separate gel the protease enzyme (−1-frame) was detected using an anti-protease antibody together with secondary antibody. The filter was stripped and re-probed as before with anti-GAPDH for lane-to-lane normalization (B). The intensity of the developed bands was quantitated (C), and the frameshift efficiency calculated for each compound at each concentration and for the DMSO control. These were normalized to the GAPDH intensities, and compared with the DMSO control.

To further characterize the enhancers as specifically affecting the efficiency of HIV-1 frameshift element we had initially tested enhancers and inhibitors isolated from the library screen in an infectivity assay with human cells infected with HIV-1, where the end point was cell viability tested with the MTS assay [38]. No significant effects were detected in this assay with the compounds found to enhance or inhibit frameshifting in the screen by ~2–fold at 20 μM. This was not surprising as the ~2-fold fold change in frameshift efficiency was likely below the limit of change required to inhibit infectivity [13]. Improvement of these compounds is ongoing. To determine whether the same pattern and degree of modulation that had been obtained in the screening programme could be reproduced with HIV-1 proteins in the 0- and –1-frames, we tested the most promising enhancers in HIV-1 infected H9 cells. Following toxicity screens the effects on synthesis of the viral protease encoded in the −1-frame compared with the 0-frame gag structural protein were determined at three time points and three concentrations of the compounds. With each compound from 1−20 μM there was an increasing concentration-dependent enhancement of viral protease compared to gag protein (1.5−2.2 fold at 20 μM) compared with solvent control (Fig 5). This was the same pattern and degree of enhancement that had been seen with the cellular screening assay and it reinforced the specificity of the enhancers for modulating –1 frameshifting within the HIV-1 element.

### Comparing enhancers of HIV-1 frameshifting with their effects on the *PEG10* element

**Fig 6:**
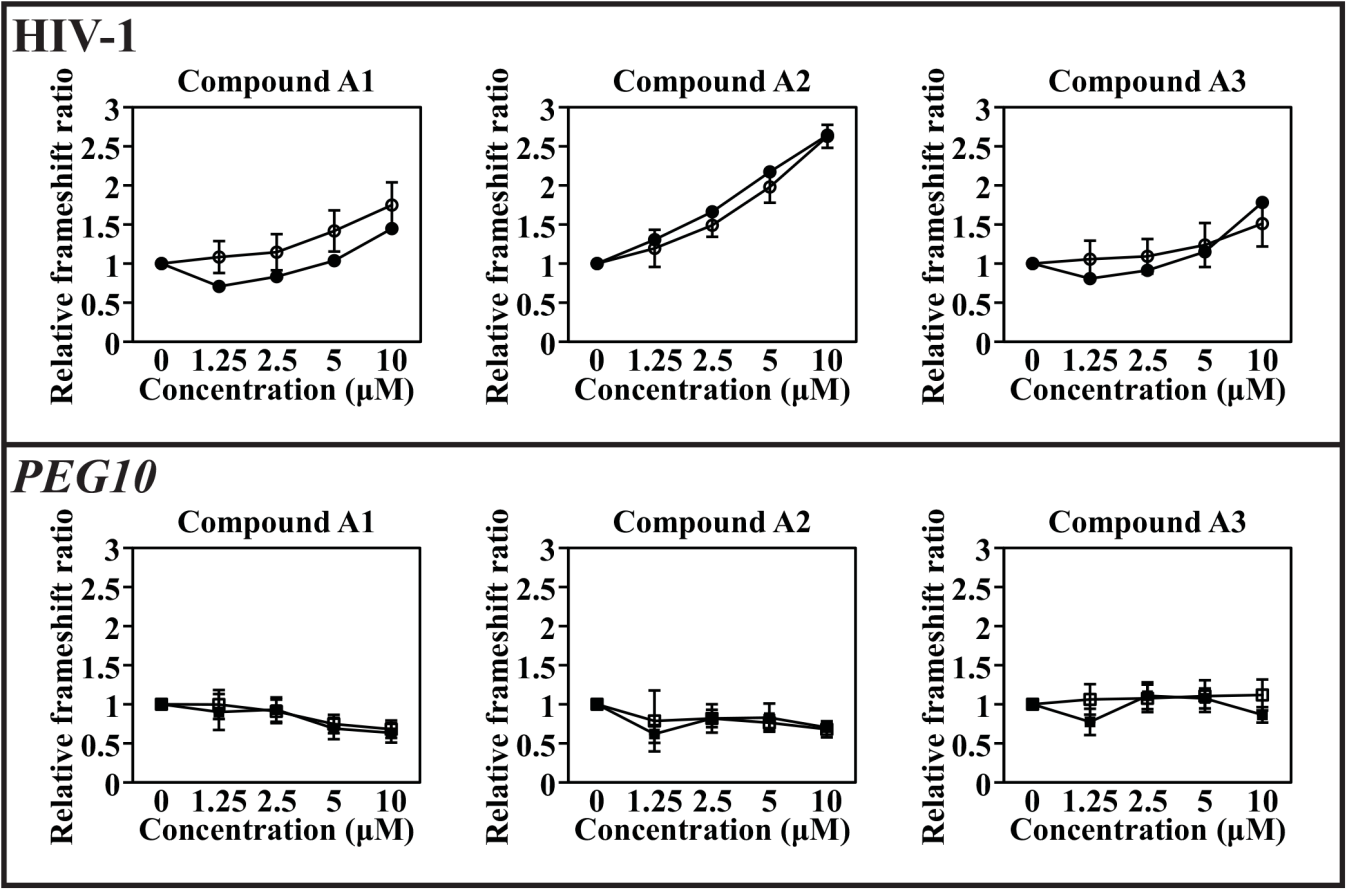
HIV-1 frameshift enhancers evaluated at both the HIV-1 and the *PEG10* frameshift elements using dual luciferase reporters and bifluorophore reporters. Effects of enhancers A1, A2 and A3 with the bifluorophore reporters (open markers), and dual luciferase reporters (closed markers) on HIV-1 frameshifting (upper panel) and *PEG10* frameshifting (lower panel) in a transient cell based assay. Results show the mean and standard deviation for quadruplicate replicates of three independent assays for the dual fluorophore assays, with the *PEG10* element tested with two biological replicates in the dual luciferase assay.

We used both a dual luciferease reporter system and a bifluorescence reporter system to test out whether the human *PEG10* frameshift element behaved differently to the HIV-1 element with the isolated enhancers. For the second reporter system, the elements and their variants were cloned between the EGFP and tdTomato bifluorescence reporters and cells transfected for transient expression (Fig 6). They are compared with the dual luciferase reporters also containing either of the two elements (Fig 6; HIV-1 above and *PEG10* below). Each compound enhanced frameshifting of the HIV-1 element in both the bifluorescent and luciferase assays and to a similar extent in each reporter system (Fig 6 upper panel). In contrast to the results with the HIV-1 element, frameshifting at the *PEG10* frameshift site was affected by only one of the compounds, A1, and was inhibited rather than enhanced with both reporter systems (Fig 6, lower panels). A2 showed a suggestion of inhibition but it was very small while A3 had no effect. This confirmed that unique features of different frameshift elements might make each of them amenable to modulation differentially by specific compounds.

## Discussion

Modulators of the HIV-1 frameshift efficiency have the potential to be specific for the HIV-1 frameshift element because of structurally unique features that distinguish it from the now well documented frameshift elements of two human genes, *PEG10* [16] and *CCR5* [19]. These two human genes have themselves quite different frameshift elements with different functional consequences. Frameshifting has been identified *in vivo* after extensive studies with *PEG10* [14,16]. The different slippery sequences, intercodons, and downstream secondary structures facilitated a frameshift efficiency that was ~2.5-fold higher for *PEG10* than for HIV-1 (Fig 1). This is despite the fact that after −1 slippage the HIV-1 sequence can make two cognate codon/anticodon pairings [39] whereas slippage allows for only one cognate re-pairing in the *PEG10* gene. The intercodon is highly conserved in HIV-1 [26], but substitution with a stop codon had a greater effect in *PEG10* decreasing frameshifting 4–5-fold compared with 2–3-fold in HIV-1. With the HIV-1 element this can be further reduced by overexpressing the decoding molecule eRF1 and this can be explained as we identified frameshifting can occur at a site within the sequence element after the intercodon has been decoded (Fig 2). Hence with the substituted intercodon the 0-frame UGA would be decoded before frameshifting. By contrast *PEG10* was insensitive to overexpession of eRF1, and it was shown to frameshift predominantly at two sites only in our heterologous system, both before the intercodon. There was however, suggestive evidence of a low frequency event after decoding of the intercodon (Table 1). Frameshifting at multiple sites with a frameshift element has recently been reported in the *dnaX* gene, and drop-off of incomplete polypeptides is inferred to occur from fidelity checks when the base pairing is non-cognate [40]. Such an outcome could contribute significantly to the large reduction in the output of the downstream reporter in the −1-frame in our *PEG10* study when the UCC intercodon is substituted with UGA. Having only one rather than two cognate re-pairings on frameshifting might destabilize the complex, allowing a significant amount of drop-off and thereby reduced frameshift efficiency.

Can compounds be isolated that modulate frameshift efficiency in HIV-1 but not in human gene *PEG10*? Using specific purpose-based cell-based assays, in this study we have screened a large small molecule library that included compounds that are desirable starting points for further lead development. Several frameshift enhancers showed no activity against the 0-frame control element or in counter screens. A primary isolate from the screen and two analogues were tested also with bifluorescent reporters (Fig 6), and they mimicked the enhancement of frameshifting seen in the luciferase assays within the HIV-1 element (Fig 3A) indicating the observed activity was independent of the reporter set. These compounds also had no effect on readthrough of a nonsense codon at a different recoding site, the CFTR W1282X UGA nonsense mutation context, found in patients with cystic fibrosis (Fig 3B). The isolated enhancers in their current configuration did not give sufficient frameshift modulation to inhibit HIV-1 infectivity significantly in cell viability assays, but the synthesis of the protease enzyme in the −1-frame was enhanced ~2-fold by these compounds in HIV-1 infected cells relative to the 0-frame gag protein. While the minimum increase in frameshift ratio needed to inhibit infectivity is not known precisely, collective studies suggest it might be at least in the order of 3-fold. Hence further chemical modifications to the isolated compounds are needed to facilitate improvement in their efficacy and potency.

While programmed −1 translational frameshifting is a rare recoding event used mainly by viruses it has yet to be exploited as a therapeutic target. Now human genes are emerging that use the same generic mechanism. PEG10 protein expression *with* frameshifting has been documented only in placenta, in amniotic membranes and at low levels in embryos, and not observed in other tissues despite extensive analysis [14,16]. Here, we tested our isolated enhancer compounds on the *PEG10* element. Two of the compounds A2 and A3 (Fig 6) that enhanced frameshifting with the HIV-1 element had no or little effect on *PEG10* frameshifting. The other enhancer, A1, modestly inhibited *PEG10* frameshifting in a concentration-dependent manner. This illustrates discovery of compounds specific for HIV-1 should be possible. Some frameshift modulators, depending on their mode of action might affect all frameshift sites however, and comprehensive testing of all human –1 frameshift sites is therefore important. The direct mechanism of action of the promising compounds we have identified is now being determined. Since they differentiate the HIV-1 and *PEG10* elements that have quite different secondary structural components (stem-loop and pseudoknot) we predict they would likely target this part of the element. Nevertheless modulating compounds could target the coding RNA at the slippery sequence, or the secondary structural element, or indeed the active center of the host human translating ribosome that has a major function of maintaining the reading frame. The sites of interaction of the A1, A2 and A3 compounds with the HIV-1 frameshift element are currently being investigated.
